# Multiple-to-Multiple Relationships between MicroRNAs and Target Genes in Gastric Cancer

**DOI:** 10.1371/journal.pone.0062589

**Published:** 2013-05-08

**Authors:** Yutaka Hashimoto, Yoshimitsu Akiyama, Yasuhito Yuasa

**Affiliations:** Department of Molecular Oncology, Graduate School of Medical and Dental Sciences, Tokyo Medical and Dental University, Tokyo, Japan; Sapporo Medical University, Japan

## Abstract

MicroRNAs (miRNAs) act as transcriptional regulators and play pivotal roles in carcinogenesis. According to miRNA target databases, one miRNA may regulate many genes as its targets, while one gene may be targeted by many miRNAs. These findings indicate that relationships between miRNAs and their targets may not be one-to-one. However, many reports have described only a one-to-one, one-to-multiple or multiple-to-one relationship between miRNA and its target gene in human cancers. Thus, it is necessary to determine whether or not a combination of some miRNAs would regulate multiple targets and be involved in carcinogenesis. To find some groups of miRNAs that may synergistically regulate their targets in human gastric cancer (GC), we re-analyzed our previous miRNA expression array data and found that 50 miRNAs were up-regulated on treatment with 5-aza-2'-deoxycytidine in a GC cell line. The “TargetScan” miRNA target database predicted that some of these miRNAs have common target genes. We also referred to the GEO database for expression of these common target genes in human GCs, which might be related to gastric carcinogenesis. In this study, we analyzed two miRNA combinations, miR-224 and -452, and miR-181c and -340. Over-expression of both miRNA combinations dramatically down-regulated their target genes, *DPYSL2* and *KRAS*, and *KRAS* and *MECP2*, respectively. These miRNA combinations synergistically decreased cell proliferation upon transfection. Furthermore, we revealed that these miRNAs were down-regulated through promoter hypermethylation in GC cells. Thus, it is likely that the relationships between miRNAs and their targets are not one-to-one but multiple-to-multiple in GCs, and that these complex relationships may be related to gastric carcinogenesis.

## Introduction

MicroRNAs (miRNAs), a class of small non-protein-coding RNAs, have been identified as a new type of gene regulator that bind to the 3'-untranslated regions (UTRs) of target mRNA, thereby resulting in mRNA degradation or the blockade of mRNA translation [1]. It is generally known that miRNA alterations are associated with tumorigenesis [1]. According to miRNA target databases, one miRNA may regulate many genes as its targets, while one gene may be targeted by many miRNAs. However, numerous studies revealed a one-to-one relationship between miRNA and its target gene. It has also been reported that multiple or a cluster of miRNAs co-operatively regulate a gene, which is related to carcinogenesis [2]–[4]. On the other hand, multiple genes are targeted by one miRNA [3], [4]. Even multiple-to-multiple relationships between miRNAs and targets have been reported by using computational analyses [5], [6]. However, there have been only a few papers experimentally validating multiple-to-multiple relationships in cancer cells.

Gastric cancer is the fourth most common human malignant disease and the second most frequent cause of cancer-related death worldwide, with an estimated one million new cases per year [7]. Gastric cancers are histologically classified into two major types, the intestinal and diffuse types [8]. Diffuse-type GC is often intractable and exhibits a poor patient prognosis. Recently, we demonstrated that loss of the Cdh1 and Trp53 functions induces diffuse type GC using mice models [9]. Although this finding will help us to develop new human gastric cancer therapies, further investigations on the molecular mechanisms underlying gastric carcinogenesis are necessary to develop other approaches for targeted therapy.

Promoter CpG island hypermethylation is one of the most common mechanisms by which tumor suppressor genes are inactivated in human cancers [10]. Recently, it has become apparent that some miRNAs are also targets of epigenetic silencing in cancers [11]. Our and other groups have previously shown that pharmacologic or genetic disruption of DNA methylation in cancer cell lines induces up-regulation of substantial numbers of miRNAs [12]–[15]. These data led to identification of candidate tumor-suppressive miRNAs whose silencing is associated with CpG island methylation. So far, methylation of miR-124 family members has been identified in colorectal cancer and in tumors of other organs [11]. In addition, the miR-34b/c cluster has a typical CpG island, and is down-regulated through frequent methylation in colorectal and gastric cancers [12]. Similarly, we found that miR-181c methylation is associated with gastric carcinogenesis via regulation of oncogenic genes *KRAS* and *NOTCH4* [13]. Down-regulation of many miRNAs through methylation simultaneously occurs in cancer cells, and may enhance multiple-to-multiple relationships between miRNAs and targets.

In this study, to validate multiple-to-multiple relationships between miRNAs and targets in cancer cells, we demonstrated that two pairs of multiple miRNAs, miR-224 and -452, and miR-181c and -340, had multiple target genes and synergistically decreased cell proliferation through regulation of their targets in human GC cells.

## Results

### 50 miRNAs were up-regulated in a GC Cell Line, KATO-III, after 5-aza-2'-deoxycytidine Treatment

**Figure 1:**
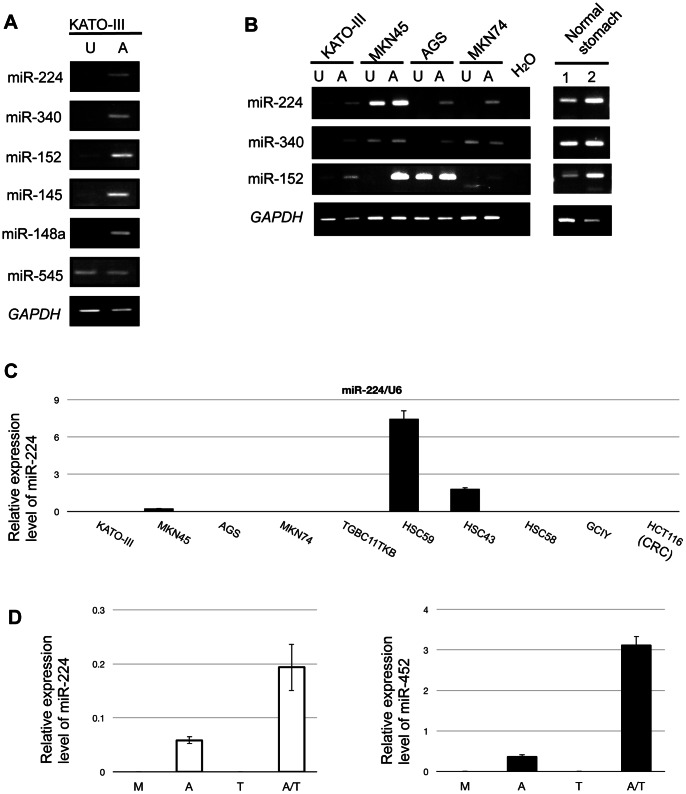
Expression patterns of human miRNAs in GC cell lines after 5-aza-CdR treatment. (**A**) RT-PCR analyses of precursor miRNAs in KATO-III cells untreated (U) or treated (A) with 5-aza-CdR. *GAPDH* mRNA expression was used as a loading control. (**B**) RT-PCR analyses of precursor miRNAs in human GC cell lines untreated (U) or treated (A) with 5-aza-CdR (5 µmol/l), and normal gastric mucosae. *GAPDH* mRNA expression was used as a loading control. (**C**) Quantitative real-time RT-PCR analysis of mature miR-224 expression in 9 GC cell lines and a CRC cell line, HCT116. (**D**) Quantitative real-time RT-PCR analysis of the mature miR-224 and -452 levels in KATO-III cells untreated (U) and treated with 0.2 µmol/l 5-aza-CdR (A), 0.3 µmol/l TSA (T), or a combination of the two drugs (A/T). M, mock.

To identify candidate miRNAs that synergistically affect their target genes, we re-analyzed our previous microarray data (GEO accession No. GSE16006) [13]. We considered that miRNA levels were up-regulated on 5-aza-2'-deoxycytidine (5-aza-CdR) treatment when the net intensities of particular 3 spots were more than 1.5-fold. Furthermore, we also selected miRNAs of which the averages were found to be increased more than 3-fold on microarray analysis. Based on these new criteria, we found that 50 miRNAs were up-regulated in a GC cell line, KATO-III (Table S1). We confirmed the up-regulation of 5 of 6 representative precursor miRNAs, that is, miR-145, -148a, -152, -224 and -340, after 5-aza-CdR treatment by RT-PCR (Figure 1A).

### TargetScan Predicted Common Target Genes of the Candidate miRNAs

**Figure 2:**
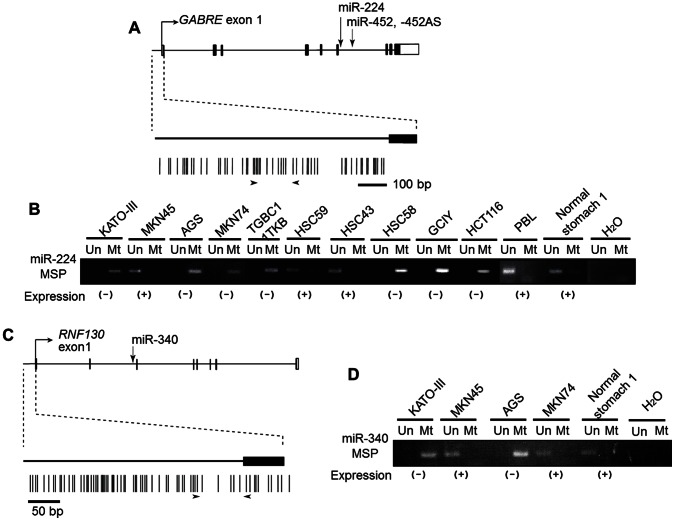
MSP analysis of miR-224/−340 in GC, CRC cell lines and normal stomach. (**A**), (**C**) Schematic representation of the miRNAs and their host genes. Filled boxes represent the exons of the host genes and blank boxes denote the untranslated regions of the host genes. The bent arrows indicate the transcription start sites of the host genes. The vertical arrows indicate the locations of miRNAs. The vertical thick lines indicate CpG sites. Arrowheads indicate the regions examined for MSP. (**B**), (**D**) MSP analyses of miR-224 and miR-340, respectively, in GC cell lines. The bands in the ‘Mt’ lanes are PCR products obtained with methylation-specific primers, and those in the ‘Un’ lanes were obtained with unmethylation-specific primers; PBL, peripheral blood lymphocyte. The expression of miRNAs is indicated under the photographs of the MSP results.

**Figure 3:**
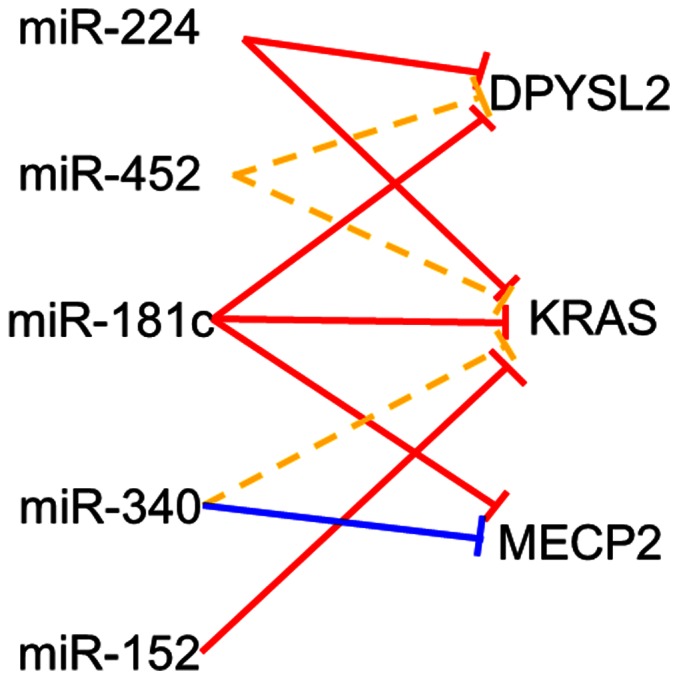
A diagram of the relationship between up-regulated miRNAs after 5aza-CdR treatment and their candidate target genes. Red lines, conserved site; blue line, conserved site in mammals; yellow lines, poorly conserved site.

To determine whether or not epigenetically regulated miRNAs have common target genes, we searched the TargetScan database (version 5.1) for their common targets. According to TargetScan, 50 miRNAs could be classified into 46 groups based on their seed sequences. TargetScan also showed that these 46 groups can target 6,460 genes. Among these target genes, we selected 13 of which expression was reported to be increased in GC in the GEO database (GEO accession No. GSE2685) or to be related to gastric carcinogenesis (Table S2). Here, we focus on four miRNAs, miR-152, -181c, -224 and -340, because we have already reported that miR-181c is epigenetically down-regulated in GC [13] and CpG islands are located in the upstream regions of three other miRNAs (Figure 2A and C, and Figure S1). Figure 2a also reveals that miR-452 is clustered with miR-224. TargetScan predicted that the five miRNAs can regulate common targets. For instance, *DPYSL2* (dihydropyrimidinase-like 2, also known as collapsing response mediator protein 2, *CRMP2*) is targeted by miR-224, -452, and -181c, *KRAS* by miR-224, -452, -181c, -340 and -152, and *MECP2* (methyl CpG binding protein 2) by miR-181c and -340, respectively (Figure 3).

### Expression of miR-224, -452, -152 and -340 Decreased on DNA Hypermethylation in GC Cell Lines

We examined the involvement of epigenetic changes in down-regulation of the miRNAs. The expression of miR-224, -340 and -152 was increased by 5-aza-CdR treatment in several GC cell lines (Figure 1B). We quantitatively analyzed mature miR-224 expression in 9 GC cell lines and a colorectal cancer (CRC) cell line. No expression of miR-224 was detected in 7 of 10 cancer cell lines (Figure 1C). We also analyzed the expression change of the miR-224/−452 cluster in KATO-III cells treated with a low dose of 5-aza-CdR (0.2 µmol/l), a histone deacetylase inhibitor, trichostatin A (TSA, 0.3 µmol/l), or a combination of these two drugs. KATO-III cells with low-dose 5-aza-CdR treatment exhibited up-regulation of the miR-224/−452 cluster, whereas TSA alone did not cause up-regulation. The miR-224/−452 cluster was synergistically up-regulated in KATO-III cells with combined 5-aza-CdR and TSA treatment (Figure 1D). These results indicate that miR-224 and miR-452 may be down-regulated through DNA methylation in GC cell lines as the same transcription unit.

It has been reported that intronic miRNAs are regulated through promoter methylation of their host genes [14], [15]. According to the results of computational analysis, the miR-224/−452 cluster and miR-340 are located in intron 6 in *GABRE* and intron 2 in *RNF130*, respectively, both of which contain dense CpG islands only in the promoter regions of their host genes (Figure 2A and C). We investigated the relationship between the expression levels of these miRNAs and the methylation status of their host genes in GC cell lines by MSP analysis. GC cell lines without miR-224 expression exhibited only methylation signals, whereas expression-positive cell lines exhibited strong unmethylation patterns (Figure 2B). A similar relationship was detected for miR-340 in GC cell lines (Figure 2D). These data indicate that expression of these miRNAs in GC cell lines may be silenced through promoter methylation of their host genes.

### Epigenetically Regulated miRNAs Might be Related to GC Cell Proliferation

**Figure 4:**
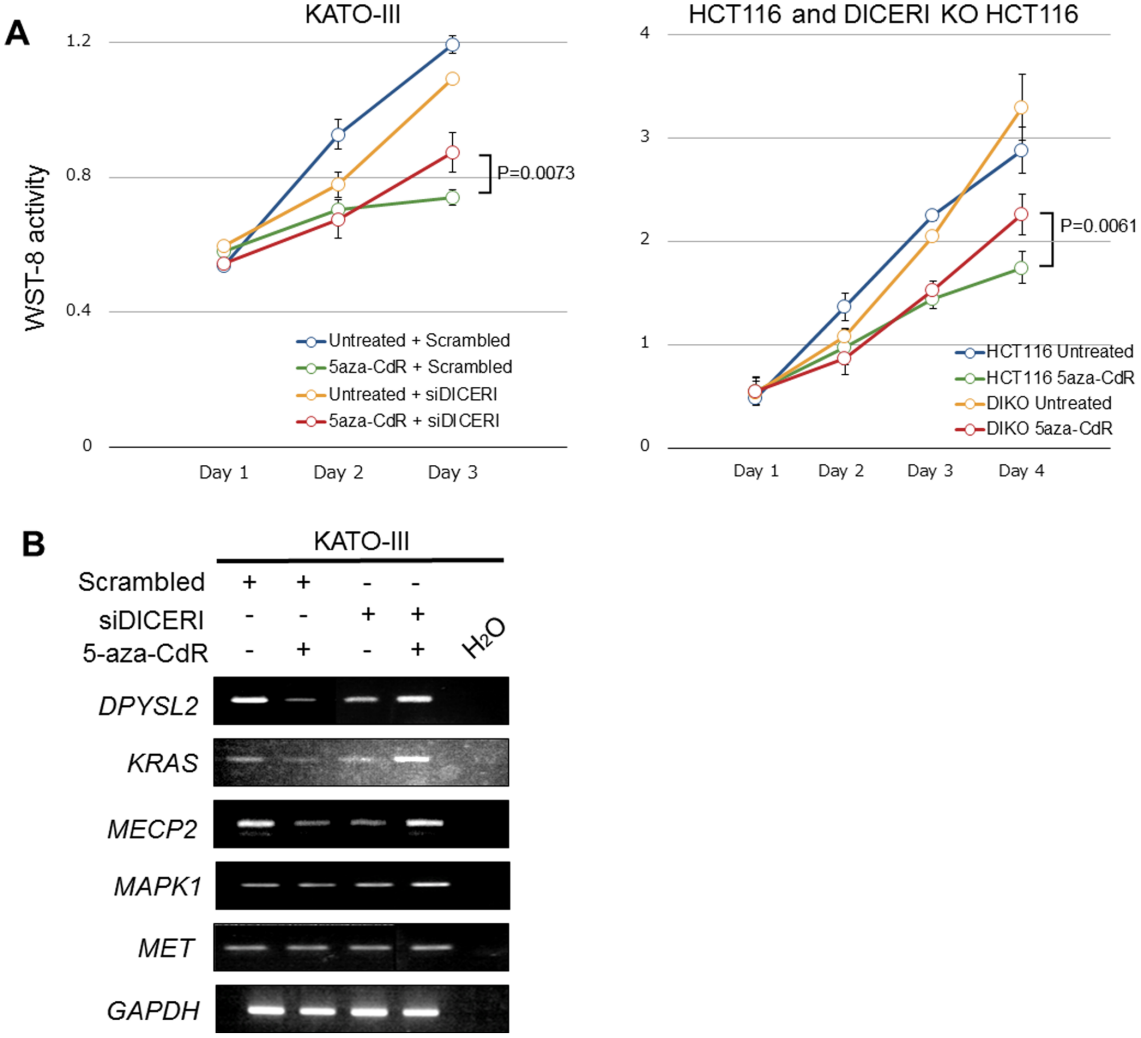
Changes of candidate target genes expression and cell proliferation after decreased DICER1 expression and/or 5-aza-CdR treatment. (**A**) The growth curves of KATO-III, HCT116 and DICER1 KO HCT116 cells. KATO-III cells were transfected with 20 nmol/l of siDICER1 or scrambled siRNA. The paired t-test was used to compare the values for the test and control samples. A value of P<0.05 was taken as significant. (**B**) Representative results of target gene expression on the RT-PCR analysis.

To determine whether epigenetically regulated miRNAs are tumor-suppressive or not, we evaluated the effect of 5-aza-CdR in siDICER1-transfected KATO-III and DICER1 KO HCT116 (D1KO) cells (Figure 4A). The untreated KATO-III and parental HCT116 cells showed decelerated proliferation after treatment with 5-aza-CdR. On the other hand, in siDICER1-transfected KATO-III and D1KO cells, the effect of 5-aza-CdR treatment on cell proliferation became weak in both cases. These results suggest that the effect of 5-aza-CdR was decreased in low or null DICER1 cells, and that epigenetically regulated miRNAs play an important role in GC and CRC cells.

In order to analyze the relationship between these miRNAs and 13 target genes shown in Table S2, we transfected KATO-III cells with siDICER1 and/or treated with 5-aza-CdR. Although, expression of 3 (*DPYSL2*, *KRAS* and *MECP2*) of the 13 genes were decreased after 5-aza-CdR treatment, the expression of these genes did not change on 5-aza-CdR treatment followed by transfection of siDICER1 (Figure 4B).

### Combinational Transfection of miR-224 and -452 Repressed GC Cell Proliferation

**Figure 5:**
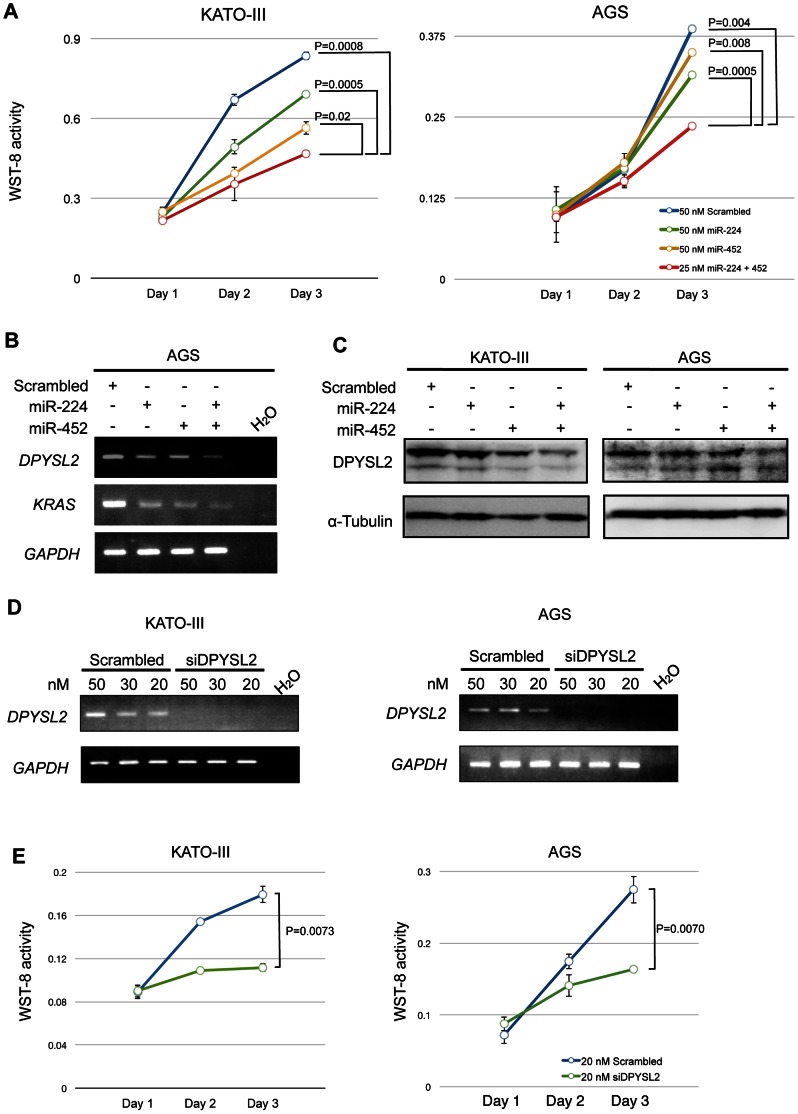
Effects of combinational transfection of miR-224 and-452 in GC cell lines. (**A**) Proliferation assaying with WST-8. KATO-III and AGS cells were seeded at 1×10^3^ and 1×10^4^ cells in 96-well plates, respectively, and cells were counted at 24, 48 and 72 h. Cultures were performed in triplicate; bars, SD. The indicated miRNAs were transfected into both cell lines either alone or in combination. (**B**) RT-PCR analysis after transfection with miR-224 and/or miR-452. The expression of target genes was analyzed 48h later by RT-PCR. (**C**) DPYSL2 protein expression after ectopic expression of miRNA mimics in KATO-III and AGS cells. The DPYSL2 protein was analyzed 72h later by Western blotting. (**D**) RT-PCR analysis of *DPYSL2* expression in KATO-III and AGS cells treated with either scrambled or *DPYSL2* siRNA. (**E**) Proliferation assaying after transfection with siDPYSL2 or Scrambled in KATO-III and AGS cells.

We transfected GC cell lines with miR-224 and/or -452 mimics as a representative of miRNA clusters, or a negative control, and then carried out water-solved tetrazolium-8 (WST-8) assays. Seventy-two hours after transfection, we observed that ectopic expression of miR-224 or -452 suppressed the growth of two cell lines, KATO-III and AGS (Figure 5A). Notably, combinational transfection of the miR-224 and -452 mimics extensively decreased the growth of the two GC cell lines (Figure 5A).

### The miR-224/−452 Cluster Co-operatively Decreased Expression of *DPYSL2* and *KRAS*

To investigate whether or not the miR-224/−452 cluster is actually related to the regulation of *DPYSL2* and *KRAS*, we analyzed the expression of *DPYSL2* after transfection of KATO-III and AGS cells with the miR-224/−452 cluster alone or together. We carried out RT-PCR and Western blot analyses. The *DPYSL2* and *KRAS* mRNA levels were decreased after transfection with the miR-224 or miR-452 mimic (Figure 5B). Interestingly, in the case of combinational transfection with miR-224 and -452, expression of these target genes was further down-regulated (Figure 5B). The down-regulation of DPYSL2 was also observed at the protein level in the two cell lines (Figure 5C). We also examined the expression levels of other five genes, *MECP2, MYC, JUNB, MUC1* and *SETDB1*, which were not shown as targets for miR-224 or -452 by TargetScan. As expected, no expressional changes of these five genes were found in KATO-III cells after transfection with miR-224 or -452 (Figure S2). These data suggest that miR-224 and -452 specifically down-regulated *DPYSL2* and *KRAS*.

### *DPYSL2* was Associated with GC Cell Proliferation

We examined the effect of knockdown of *DPYSL2*, which was shown to be a target of the miR-224/−452 cluster, on cell proliferation. The transfection of *DPYSL2* siRNA clearly decreased the levels of the *DPYSL2* transcripts (Figure 5D), and inhibited the growth of AGS and KATO-III cells 72 hours after the knockdown of *DPYSL2* (Figure 5E), indicating that DPYSL2 has an oncogenic activity.

### Combinational Transfection of miR-340 and -181c Repressed GC Cell Proliferation, and Induced Down-regulation of *KRAS* and *MECP2* Expression

**Figure 6:**
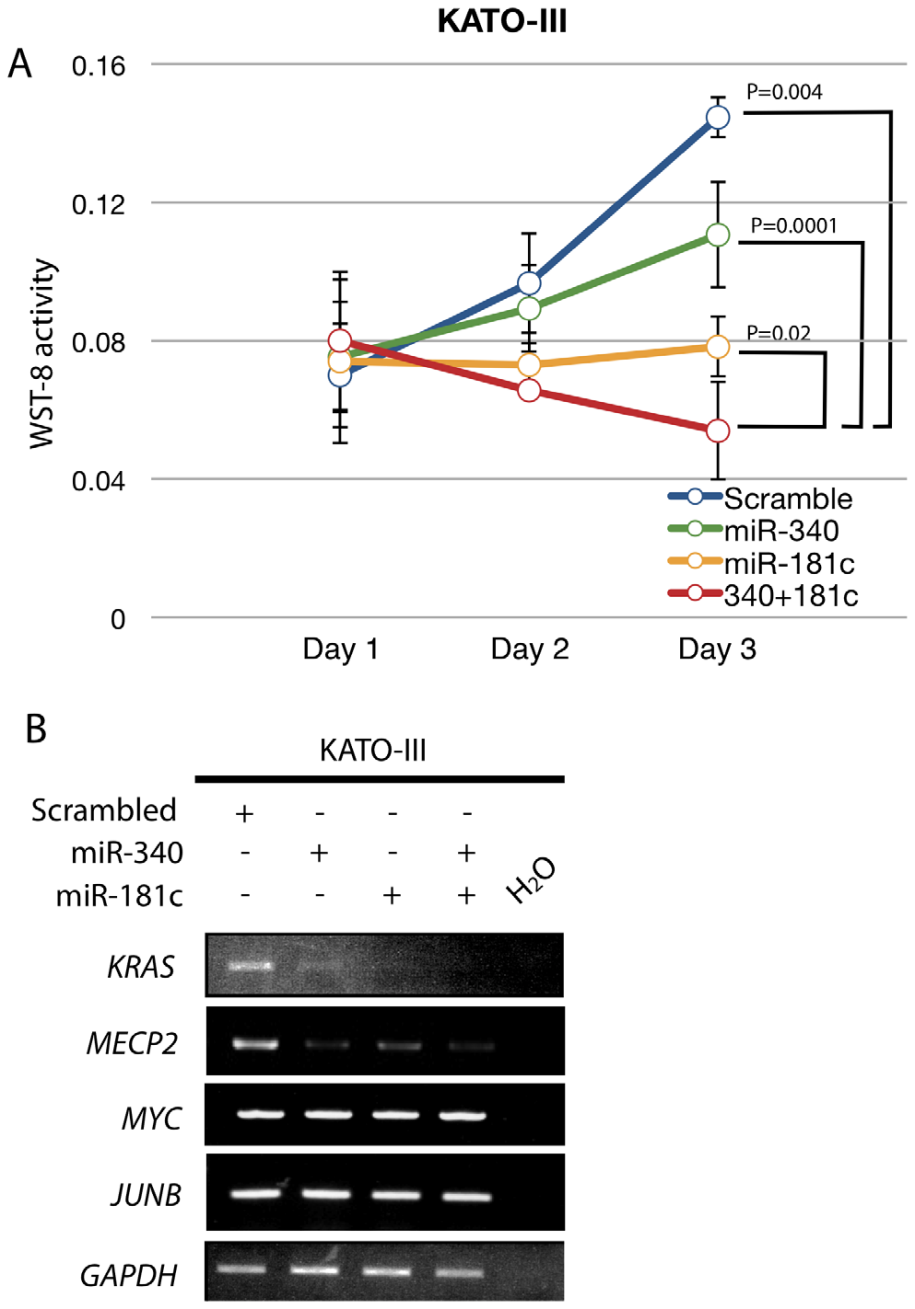
Effects of combinational transfection of miR-340 and -181c in GC cell lines. (**A**) Proliferation assaying after transfection with miR-340 and/or miR-181c mimics in KATO-III cells. (**B**) Changes in gene expression after ectopic expression of miR-340 and/or miR-181c.

As a second example of multiple-to-multiple relationships between microRNAs and target genes, we analyzed the relationship between miR-340/−181c and *KRAS/MECP2.* When miR-340 and miR-181c were transfected into KATO-III cells, proliferation was synergistically down-regulated by two miRNAs (Figure 6A). To determine whether or not epigenetically regulated miR-340 and miR-181c co-operatively affect their targets, we analyzed the mRNA levels of *KRAS* and *MECP2.* On RT-PCR analysis, *KRAS* and *MECP2* were found to be down-regulated by miR-340 and miR-181c alone, or combinational transfection in KATO-III cells (Figure 6B). As for the four genes, *MYC, JUNB, MUC1* and *SETDB1*, showing no predicted sites for these two miRNAs by TargetScan, the expression levels were not changed in this study. Representative data are shown in Figure 6B. Thus, the effects of miR-340 and -181c may be specific to their common target genes as well as miR-224 and -452.

### Expression and Methylation Status of miR-224 and -340 in Primary GC Cases

**Figure 7:**
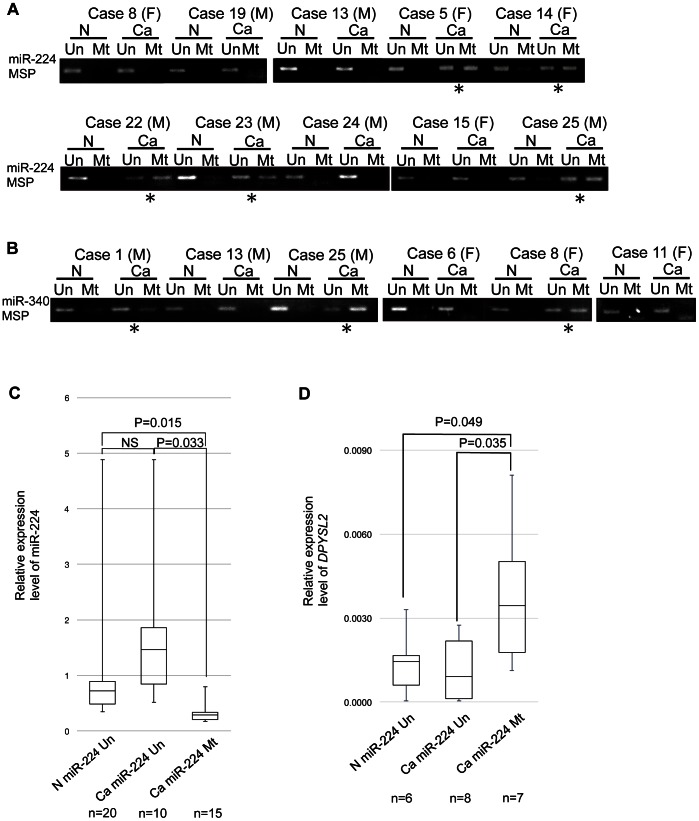
Methylation analysis of miR-224/−340 and expression of *DPYSL2* in human gastric cancer tissues. Representative results of miR-224 (**A**) and miR-340 (**B**) MSP analyses in primary GC tissues. The bands in the ‘Mt’ lanes are PCR products obtained with methylation-specific primers, and those in the ‘Un’ lanes were obtained with unmethylation-specific primers. ‘Ca’ and ‘N’ denote primary GCs and paired non-cancerous tissues, respectively. ‘F’ and ‘M’ denote female and male. Black stars indicate samples in which the aberrant hypermethylation of CpG islands was detected. (**C**) Comparison of the miR-224 methylation status and miR-224 levels in gastric tissues. The relative levels of miR-224 expression in gastric tissues were determined by real-time RT-PCR and then compared with the miR-224 methylation status in gastric tissues: N miR-224 Un (non-cancerous without miR-224 methylation; n = 20), Ca miR-224 Un (GC tissues without miR-224 methylation; n = 10), and Ca miR-224 Mt (GC tissues with miR-224 methylation; n = 15). The two-sample t-test was performed to examine the difference between two groups. (**D**) Comparison of the miR-224 methylation status and *DPYSL2* mRNA levels in GC tissues. The relative levels of *DPYSL2* expression in gastric tissues were determined by real-time RT-PCR, normalized as to *GAPDH*, and then compared with the miR-224 methylation status in gastric tissues: N miR-224 Un (non-cancerous without miR-224 methylation, n = 6), Ca miR-224 Un (GC tissues without miR-224 methylation, n = 8), and Ca miR-224 Mt (GC tissue with miR-224 methylation, n = 7). The boxes indicate the 25th and 75th percentiles, the horizontal lines inside the boxes indicate the medians and the bars indicate the 10th and 90th percentiles. NS, not significant.

We examined the methylation status of miR-224 and -340 in primary GC cases. Methylated patterns of miR-224 were detected in 15 of 26 (57.7%) primary GC tissues (Figure 7A and Table 1). Paired non-cancerous gastric mucosae hardly exhibited a methylation pattern of miR-224. Next, we quantitatively examined the miR-224 levels in primary GC tissues and corresponding non-cancerous mucosae by TaqMan RT-PCR. A significant reduction of miR-224 expression in GC tissues was observed in methylation-positive cancer cases compared with in methylation-negative ones and non-cancerous gastric mucosae (Figure 7C).

**Table 1:** The results of MSP analysis in primary GCs.

Case #	miR-224/452	miR-340
1	U	**M**
2	**M**	U
3	**M**	U
4	**M**	U
5	**M**	U
6	U	U
7	U	U
8	U	**M**
9	**M**	U
10	**M**	**M**
11	**M**	U
12	U	U
13	U	U
14	**M**	U
15	U	U
16	**M**	U
17	**M**	U
18	U	U
19	U	U
20	**M**	U
21	**M**	U
22	**M**	U
23	**M**	U
24	U	U
25	**M**	**M**
26	U	U

We further analyzed the *DPYSL2* mRNA levels in comparison with the methylation status of miR-224 in GC tissues: GCs with miR-224 methylation (Ca miR-224 Mt), GCs with miR-224 unmethylation (Ca miR-224 Un), and non-cancerous tissues with unmethylation (N miR-224 Un). The *DPYSL2* mRNA level in the “Ca miR-224 Mt” group was significantly higher than those in the “N miR-224 Un” and “Ca miR-224 Un” groups, p = 0.049 and p = 0.035, respectively (Figure 7D). Thus, there is a correlation between the methylation status of miR-224 and *DPYSL2* expression in GC tissues.

The miR-340 methylation frequency was relatively low in the primary GC tissues tested (4 of 26, 15.4%) (Figure 7B and Table 1), whereas none of 26 paired non-cancerous gastric mucosae exhibited apparent methylation patterns of miR-340. As for miR-152 methylation analysis, we tried three primer sets designed in the upstream region of miR-152 containing CpG islands (Figure S1), but none of them completely matched miR-152 expression on MSP analyses (data not shown).

## Discussion

Although it has been reported that the expression of some miRNAs is decreased in several cancers through DNA methylation, most of the reports described that the relationship between aberrant expression of miRNAs and its target genes was one-to-one, one-to-multiple or multiple-to-one. To examine the possibility of multiple-to-multiple relationships between miRNAs and targets in cancer cells, we focused on two combinations of miRNAs in GC cells, the miR-224/−452 cluster, and miR-181c and -340, in this study. We found that the two sets of miRNAs, miR-224 and -452, and miR-181c and -340, had multiple target genes, *DPYSL2* and *KRAS*, and *KRAS* and *MECP2*, respectively, and synergistically decreased cell proliferation in human GC cell lines. It is notable that an oncogene, *KRAS* [16], was found to be targeted by four miRNAs, although candidate binding sites of the four miRNAs are different in the 3'-UTR of *KRAS* (TargetScan). We previously reported that miR-181c down-regulated *NOTCH4* too [13]. Thus, multiple-to-multiple relationships between miRNAs and targets were indicated not only by database analyses but also by transfection experiments involving human cells.

miR-224- and/or -340-expression-negative GC cell lines exhibited hypermethylation signals on MSP analysis and the expression of miR-224 and -340 restored on demethylating agent treatment. Furthermore, hypermethylation of miR-224 and -340 was more frequently observed in primary GCs than corresponding noncancerous mucosae. In miR-224 methylation-positive cases, expression of miR-224 was significantly lower than in methylation-negative ones. These data strongly indicate that aberrant DNA methylation is one of the key mechanisms underlying down-regulation of miR-224 and -340 in GC cells.

We showed that inhibition of miRNA processing by siDICER1 transfection or using DICER1 knockout cells decreased the effect of 5-aza-CdR, that is, decreased cell proliferation and down-regulation of target genes, in GC and CRC cells. It has been reported that the abundance of DICER1, the enzyme that catalyzes the final step of miRNA maturation, is directly associated with tumor progression [17]. These results suggest that aberrant regulation of miRNA maturation contributes to GC and CRC formation.

We found that the miR-224/−452 cluster was aberrantly down-regulated in GCs through hypermethylation. Aberrant expression of miR-224 has also been reported in other tumors. Expression of miR-224, let-7f and miR-516a is decreased in ovarian cancer, and they synergistically regulate expression of kallikrein-related peptidase 10 (KLK10) [18]. miR-224 is down-regulated in methotrexate-resistant CRC cell lines compared with in sensitive cells [19]. Here we also revealed that the methylation status of miR-224 was correlated with the *DPYSL2* level in human GCs. Taken together, miR-224 plays an important role as a tumor-suppressive miRNA in GC as well as in several other cancers. In contrast, miR-224 is up-regulated in hepatocellular carcinomas [20] and medulloblastomas [21] compared with in the normal tissues. Thus, further studies are required to clarify the role of miR-224 in carcinogenesis.

We investigated the common targets of epigenetically down-regulated miRNAs. *DPYSL2* was shown to be down-regulated by miR-224 and -452. DPYSL2 plays an important role in the establishment of neuronal polarity [22]. DPYSL2 is also involved in pathways that regulate the proliferation of non-neuronal cells through its phosphorylation by regulatory proteins. DPYSL2 undergoes dynamic phosphorylation changes in response to contact inhibition-induced quiescence and hyperphosphorylation of DPYSL2 occurs in a tumor [22]. Although the role of DPYSL2 in GCs is not clear, our siRNA-based knockdown of DPYSL2 expression induced a reduction of proliferation in the two GC cell lines, suggesting oncogenic activity of DPYSL2.

In summary, our findings indicate that multiple-to-multiple relationships between miRNAs and target genes really exist in GC. It is likely that aberrant methylation decreases the expression of multiple tumor-suppressive miRNAs, such as miR-224, -452, -340 and -181c, which then induce over-expression of multiple oncogenic genes, like *KRAS*, *DPYSL2*, and *MECP2*. These abnormal multiple-to-multiple relationships between miRNAs and targets would be one of the important mechanisms underlying gastric carcinogenesis. It is, therefore, highly possible that epigenetic drugs may normalize the expression of not only tumor-suppressive genes but also multiple tumor-suppressive miRNAs, resulting in decreases of the abnormal multiple-to-multiple relationships between miRNAs and targets, and thus may become excellent therapeutic drugs against cancer.

## Materials and Methods

### Ethics Statement

Written informed consent was obtained from all subjects, and the ethics committee of Tokyo Medical and Dental University School of Medicine approved this research.

### Cell Lines and Tissue Samples

We studied 9 GC cell lines (KATO-III, MKN45, AGS, MKN74, TGBC11TKB, HSC59, HSC43, HSC58 and GCIY), 2 CRC cell lines (HCT116 and *DICER1* knock out HCT116 [23]), and 26 primary GC cases. MKN45, MKN74, TGBC11TKB and GCIY were purchased from RIKEN cell bank, and KATO-III and AGS were from ATCC (American Type Cell Collection). HSC59, HSC43 and HSC58 were obtained from Dr. Kazuyoshi Yanagihara [24], [25]. KATO-III, MKN45, MKN74, HSC59, HSC43 and HSC58 were grown in RPMI 1640, and AGS, TGBC11TKB, GCIY and the two CRC cell lines in Dulbecco’s modified Eagle’s medium, minimal essential medium or McCoy’s 5A, supplemented with 10% fetal bovine serum. Surgically resected specimens from 26 primary GC patients were randomly obtained from the Affiliated Hospital of School of Medicine, Tokyo Medical and Dental University.

### In silico Analysis

We referred to the GEO database for miRNA and gene expression profiles (GEO accession No. GSE16006 and GSE2685). We also used miRNA target database “TargetScan”.

### Drug Treatment of Cells and RNA Extraction

For demethylation studies, cells were daily treated with 5 µmol/l 5-aza-CdR (Sigma–Aldrich, St Louis, MO) for 72 h. We also treated cells with 0.3 µmol/l TSA alone, and with a combination of 0.2 µmol/l 5-aza-CdR and TSA. Total RNA was isolated by using Trizol reagent (Invitrogen, Carlsbad, CA) or a miRNeasy mini kit (Qiagen, Hilden, Germany).

### Quantitative Real-time Reverse Transcription–polymerase Chain Reaction

Real-time reverse transcription–polymerase chain reaction (RT-PCR) analyses were carried out using a StepOne Real-time PCR System (Applied Biosystems, Foster City, CA), EagleTaq Master Mix with ROX (Roche, Mannheim, Germany), a TaqMan Reverse Transcription kit (Applied Biosystems), and TaqMan miRNA assays (Applied Biosystems), according to the manufacturers’ instructions. The expression levels of miRNA were calculated based from the amount of target miRNA relative to that of RNU6B as a control to normalize the initial input of total RNA.

### Methylation Analysis

Bisulfite treatment of DNA was performed with Methylamp (Epigentek, Brooklyn, NY). Methylation-specific polymerase chain reaction (MSP) analyses were performed as described previously [26]. The primer sequences and PCR product sizes are shown in Table S3.

### Synthetic miRNA Transfection

KATO-III and AGS cells were transfected with a Precursor Molecule mimicking miR-224, -452, -340 or -181c, or scrambled sequence miRNA (Sigma) to give a final concentration of 25-50 nmol/l by using an electroporator, Neon (Invitrogen), according to the manufacturer’s instructions. At 24–72 h after transfection, cells were harvested for RT-PCR or Western blot analysis.

### Cell Proliferation Assay

miR-mimic-transfected KATO-III and AGS cells were plated at 1×10^3^ or 1×10^4^ cells per well on 96-well plates. Cell proliferation was evaluated on days 1–4 after transfection by determining the number of cells with cell proliferation reagent WST-8 (Dojindo Molecular Technologies, Inc., Mashikimachi, Japan), according to the manufacturer’s instructions.

### miRNA Target Prediction and Western Blotting

The predicted targets of miRNAs and their target sites were analyzed using TargetScan. The mRNA expression levels of the predicted targets in transiently transfected cells were analyzed 24 h after transfection by RT-PCR. Western blot analyses were performed as described previously [26]. The primary antibody used was rabbit anti-DPYSL2 (1∶500; #9393, Cell Signaling Technology, Danvers, MA). We used mouse anti-α-tubulin (1∶1000; sc-8085, Santa Cruz Biotechnology) as an internal control for Western blotting. The secondary antibodies used were alkaline phosphatase-conjugated anti-rabbit IgG and anti-mouse IgG (1∶2000; Bio-Rad Laboratories, Hercules, CA). Blots were developed with ImmunoStar AP Substrate (Bio-Rad Laboratories).

## Supporting Information

Figure S1 **Schematic representation of the** ***COPZ2*** **region containing miR-152.** Filled boxes represent the exons of *COPZ2*A and a blank box denotes the untranslated region of *COPZ2*. A bent arrow indicates the transcription start site of *COPZ2*. A vertical arrow indicates the location of miR-152. Vertical lines indicate CpG sites. Arrowheads indicate the regions examined for MSP.
(TIFF)

Figure S2 **Effects of transfection of miR-224 and -452 in KATO-III cells.** RT-PCR analyses after transfection with miR-224 and/or -452. The expression of target genes was analyzed 48h later by RT-PCR. miR-224 and -452 specifically down-regulated *DPYSL2* and *KRAS*, but not other five genes examined, which are consistent with the results of database analysis (Figure 3).
(TIFF)

Table S1 **Expression profiling of human miRNAs in KATO-III cells after 5-aza-CdR treatment.**
(XLSX)

Table S2 **List of target genes of which expression we examined by RT-PCR after treatment with or without 5-aza-CdR, and transfection with 20 nmol/L of siDICER1 or scrambled siRNA.**
(XLSX)

Table S3 **Sequences of primers used in this study.**
(XLSX)
