# Supplementary figures and images for: Multiple-to-Multiple Relationships between MicroRNAs and Target Genes in Gastric Cancer
Source: PLoS One. 2013 May 8;8(5):e62589. doi: 10.1371/journal.pone.0062589 (PMC3648557; doi:10.1371/journal.pone.0062589)

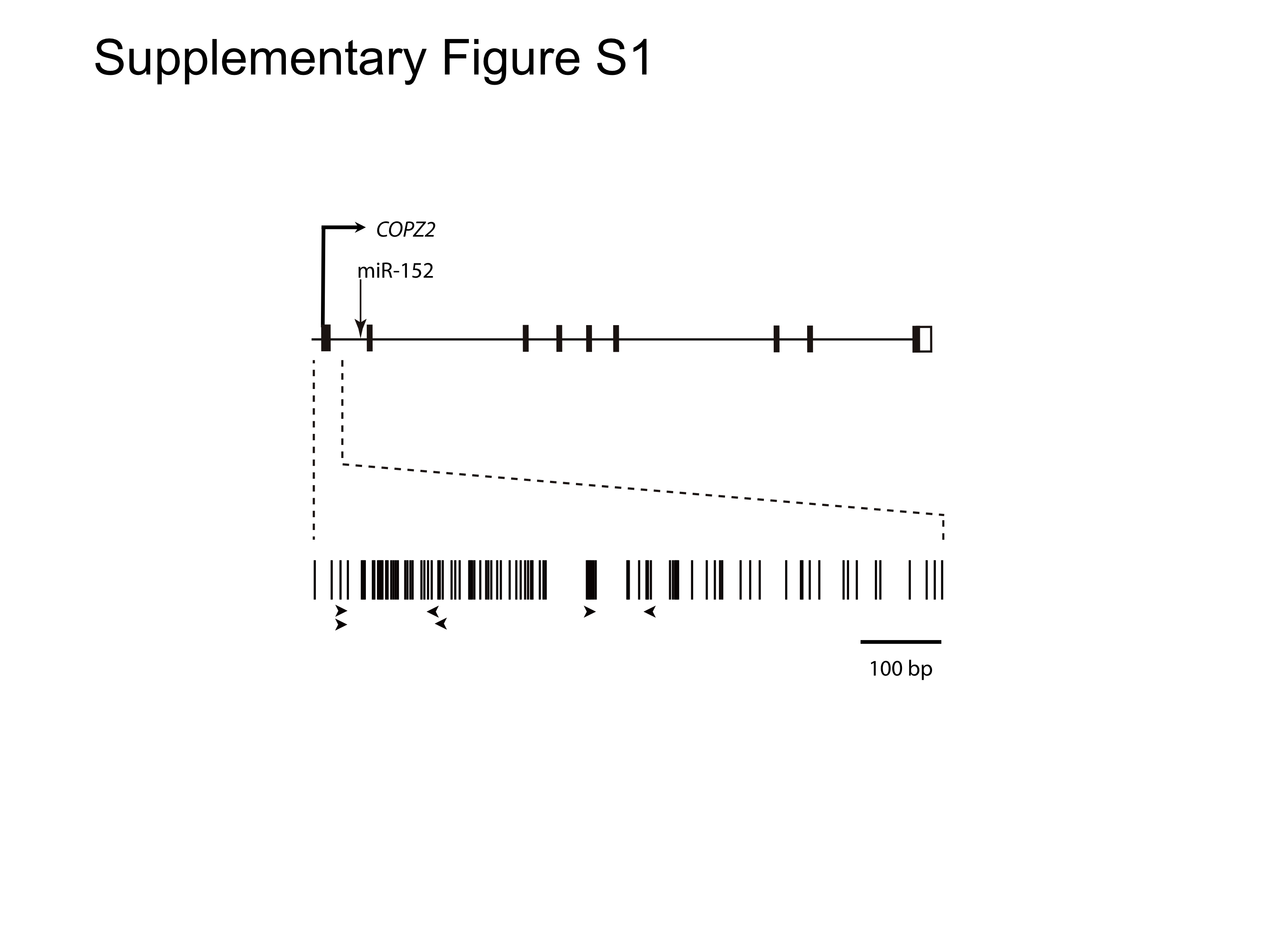

Supplement: Figure S1 — Schematic representation of the COPZ2 region containing miR-152. Filled boxes represent the exons of COPZ2A and a blank box denotes the untranslated region of COPZ2. A bent arrow indicates the transcription start site of COPZ2. A vertical arrow indicates the location of miR-152. Vertical lines indicate CpG sites. Arrowheads indicate the regions examined for MSP. (TIFF) [file pone.0062589.s001.tiff]
